# Editorial Gossip

**Published:** 1837

**Authors:** 


### III.—ORIGINAL.

### THE WESTERN JOURNAL.

*E Sylvis Nuncius.*

CINCINNATI, OHIO, JANUARY, 1838.

### Editorial Gossip.

*Compliments of the Season.*—-Writing down Anno Domini "1838," reminds us that the present calendar year is expiring, and that this number of our *quarterly* will go forth on the first of January. Let it carry, then, the "*Compliments of the Season*" to all our readers; with the hope, that they have made such collections from their rescued and grateful patients, as will enable them to keep warm fires and lamps well filled with oil. Without these important aids, they will neither be able to discover nor relish the various beauties which adorn our Journal; nor write out their summer and autumnal experience for its future pages; nor examine their private pupils; nor digest schemes of professional renown; nor cast up their uncollected debts:—And he who is obliged to pretermit these important duties, deserves the sympathy of the profession.

*Our subscription list.*—We are happy to be able to say, that our subscription list is steadily augmenting—*ergo*, the thirst for sound knowledge and late intelligence, is increasing in the *West!* This may not be a very philosophical deduction, but it makes up by its pleasantness for what it may lack in logic. We began this paragraph, under a feeling of self esteem; but so nearly is that sentiment allied to self interest, that our acquisitiveness has become excited, and suggests to us the propriety of reminding our subscribers, that by the terms of our prospectus, the subscription price of our Journal is four dollars a volume, if not paid within the publishing year. We do not expect to realize a profit on its publication; but we must make it support itself. But to come to the matter with great frankness, some early remittances would be acceptable. A three dollar bank note, of any bank in the State to which a subscriber belongs, may be remitted by mail, at our risk.

*Professor Parker.*—We are happy to announce the return of our colleague from his professional voyage to London, Paris and Rome. He is now in the active discharge of his duty, as Professor of Surgery in the Cincinnati College.

*Materiel for Instruction.*—The purchases made by Prof. Parker in Paris and London, and by Prof. Rogers in Baltimore and Philadelphia, of books, apparatus and preparations, have greatly augmented our means of instruction. Thus, by gradual accretion, our magazines of the MATERIEL are becoming adequately filled; and that, too, without any extraneous assistance.

*Number of Pupils.*—The present session has brought us a class of 122 pupils. Our first class consisted of 66, the second of 85, the third of the number just named. Thus our growth is sound, regular and encouraging. An increase at the same ratio, for the next session, will give us 175; a lesser increase for the following year will bring us to 200; which are as many as professors, not delivered over to the propensity of *covetiveness*, would desire; for they are as many as can be *advantageously* taught. We trust that our distant friends will find in these facts, an evidence of our prosperity, and a guaranty for that kind of future, which they may have hoped was in store for us.

Let us for a moment compare this progress with that of other schools in the West. The first medical professors of Transylvania were appointed in 1815. In 1816-17 and 1817-18 they had regular lectures, and drew pupils from other States than Kentucky. The latter session was attended by 20, and in the spring a medical Commencement was held. In 1818-19 the lectures were suspended. In 1819-20 they were resumed, on the basis of pecuniary aid from the people of Lexington. The class, then the fourth, consisted of 38. Next year it amounted to 94, and the next—the sixth—to 138. The Legislature of the State, meantime, had given $5000 to the University. Thus, by the aid of both the city and state, Transylvania, at the end of seven years, had but a few more pupils than the Cincinnati College now has, in its third session. Again: The Medical College of Ohio, in this city, began in 1820, with 20 pupils, and in 1830 had precisely the number which, now, in the third year, belongs to our Institution; the Legislature, in 1826, having made it a liberal endowment. These statistics, from which there can be no appeal, demonstrate that our growth is every way auspicious.

*Elements of Pathological Anatomy.*—Professor Gross is about to commit to the press, a work on Pathological Anatomy, to serve as an elementary treatise for office pupils, and a text book for those in attendance on lectures. It will be comprised in one octavo volume, and appear in the course of next spring.

*Our Alma Mater.*—We hear it reported, that the session of the University of Pennsylvania has been extended to five months, and will not terminate till the first of April. This is worthy of the great and governing school of the New World; and what with humility, but earnestness, we have been urging for several years. Her example will no doubt be followed ere long by other institutions. If all would simultaneously extend their respective sessions a month, an immediate impulse would be given to the medical profession throughout the United States.

*British Scientific Association.*—Prof. Parker, who was present, in Sept., at the yearly meeting of the British Association for the advancement of Science, in Liverpool, speaks in the highest terms of the exercises. We learn from the Boston Medical and Surgical Journal, that Professor Warren, of Harvard, was also there, and read a paper on the similarity of the skulls found in the ancient mounds of North America to those of the Peruvians. Prof. W. will spend a year in Europe. Why cannot the physicians and other scientific men of the United States form and sustain an association like that of Great Britain? Its advantages and pleasures would be manifold, and we doubt not that its meetings would be well attended.

*Lectures on Animal Magnetism, Apparitions and Witchcraft.*—One of our physicians, who is generally on the look out for an opportunity to make a speech, in the absence of any useful subject for a public lecture, lately bethought himself of Witchcraft, Second Sight, Ghosts and Animal Magnetism, and has just finished a series of four lectures on those instructive and important topics. We understand that he magnetized not a few of his auditors into sound sleep, but have not heard that any of them acquired the true somnambulic inspiration. It is probable that they have obtained such a degree of *Clairvoyance* as will enable them to distinguish ghosts and witches from all other beings, especially if they should take a second sight. We presume the gentleman has kept magic, astrology, palmistry and coscinomancy in reserve for next winter.

*Exhortation to gentility of style.*—Our friend, the Editor of the Philadelphia "Eclectic Journal of Medicine," in his November number, has read us a lecture for calling things by their right names. Now, this is in violation of all the laws of nomenclature and classification. Suppose a naturalist were to call "dog fennel" by the name "rose," or an "ermine" by that of "skunk," would not all distinctions be confounded? If we wrote in a certain style of a certain *quassi* professor, who in his floatings floated, *ex necessitate*, from this city to some other, we wrote according to the *fact*, and were not at liberty to employ any but appropriate terms. If some of these were coarse, they might, to use the language of Junius, be unworthy of *us*, but not inapplicable to the individual to whom they were applied. Our friend of the Journal of Medicine, was once a straight-forward, up-and-down Tennessee Backwoodsman, and could have endured, without flinching, the utterance, upon a contemptible object, of whatever epithets might have been necessary to a just delineation. We fear that the taste of our friend is losing its backwoods simplicity, by long residence in a great city. It is flattering to us, however, that he still retains an affection for the West, and is willing to read homilies of gentility and refinement to his benighted and half civilized brethren of the Valley of the Ohio. We hope that having done this, he will turn his attention to the western adventurer, whom he has volunteered to blow up into a great man, and try to improve *his* language a little. He who spells hæmorrhage in *four* different modes on one page, and winds up with a *flannen* roller, needs the tuition of our friend or some other school-master, and we hope, for the honor of the institution to which he is now attached, will receive it. Our friend of the Journal has charged us with "virulence of abuse." Now, there *can* be no abuse as long as facts only are stated. Our friend then virtually tells his readers, that we have not confined ourselves to facts! But how does he know this? Was he in Cincinnati during the sojourn of *his* friend among us? Is he prepared to point out a single statement made by us that is not literally true? If so, we invite him to do it. He shall have a reply, containing our proofs, to put in his Journal, that its readers may see the difference between our averments and the testimony under which they were made. That is the way to disgrace us. In conclusion, we must express the regret, that we are so remote from our friend, that we cannot have the advantage of consulting him on our style, and of acquiring those refinements of speech, which it may be conjectured, are fostered only in large cities; we shall, however, continue to be constant readers of his valuable journal, and may hope, in due time, to know something of the language, in which *les gens du Bel air* express themselves.

*British Journals.*—By arrangements recently made, we shall hereafter receive regularly the following Journals—Medico-Chirurgical Review, British and Foreign Medical Review, Lancet, London Medical Gazette, Edinburgh Medical and Surgical Journal, Records of General Science, London and Edinburgh Philosophical Magazine, and Scientific Memoirs. The Lancet and Gazette are weekly publications, and will reach us, soon after they appear, by the packets and mail. Thus it will be in our power to make an early selection of such things as are adapted to the wants of our readers.

*Books and Pamphlets for Review.*—The great length of our article on Animal Magnetism has excluded a notice of several works, sent us by authors and publishers, to whom we beg leave to return our thanks. They will receive attention in the next number.

*To Correspondents.*—Several recent communications are on file, some of which *may* be published, perhaps; but a part of them are composed of matters, which, although good in themselves, are not sufficiently important or original to justify their publication. Such is not the case with the Meterological Tables for the calendar year 1836, sent us by our ingenious and scientific friends, Professor Ray, of the Woodward College in this city, and President Williams, of the Springfield High School, in this State. But we have withheld them, in hope of being able, for the purpose of comparison, of uniting with them the statements of other meteorologists in the Valley of the Mississippi; and we now respectfully solicit such communications not only for the year 1836, but that which is now about to expire. The whole will be arranged into a table, and published in our April number.

*Professional Fees.*—We are anxious to collect *data* for an estimate of the comparative fees of physicians, surgeons and accoucheurs, in the different States of the West and South West, and will be thankful for accurate information on this subject. The charge for a visit in town, and the rate of charging a mile, the compensation for a number of the common operations in surgery, and the fee in obstetric cases, will be wanted; and every communication should state whether the physician furnishes and charges for the medicines which are prescribed, and whether the charges for surgical and obstetrical operations are followed by other charges for visits. A digest of the whole cannot fail to be interesting to the profession. We should like to receive the information in time for our next number; that is, in the course of the ensuing two months. Except when it is forbidden, the name of each contributor will be quoted as authority. As the postage on the numerous letters which we hope to receive on this subject, would, in the aggregate, be a heavy charge on the Journal, while distributed among their authors it would be a trifle to each, we shall not be displeased to see their letters post paid; unless, indeed, they should happen to enclose a year's subscription; or what would be still more acceptable, an account for publication of some of the "wonderful cures" which they have performed, even without the stimulus of large fees.

